# Early-Life Overweight Trajectory and CKD in the 1946 British Birth Cohort Study

**DOI:** 10.1053/j.ajkd.2013.03.032

**Published:** 2013-08

**Authors:** Richard J. Silverwood, Mary Pierce, Rebecca Hardy, Claudia Thomas, Charles Ferro, Caroline Savage, Naveed Sattar, Diana Kuh, Dorothea Nitsch, National Survey of Health and Development Scientific and Data Collection Teams

**Affiliations:** 1 Department of Non-Communicable Disease Epidemiology, Faculty of Epidemiology and Population Health, London School of Hygiene and Tropical Medicine, London, United Kingdom; 2 MRC Unit for Lifelong Health and Ageing, University College London, London, United Kingdom; 3 Population Health Research Centre, Division of Population Health Sciences and Education, St George's, University of London, London, United Kingdom; 4 Department of Renal Medicine, Queen Elizabeth Hospital, Birmingham, United Kingdom; 5 School of Immunity and Infection. College of Medical and Dental Sciences, University of Birmingham, Birmingham, United Kingdom; 6 BHF Glasgow Cardiovascular Research Centre, University of Glasgow, Glasgow, United Kingdom

**Keywords:** Childhood obesity, chronic kidney disease, estimated glomerular filtration rate

## Abstract

**Background:**

Few studies have examined the impact of childhood obesity on later kidney disease, and consequently, our understanding is very limited.

**Study Design:**

Longitudinal population-based cohort.

**Setting & Participants:**

The Medical Research Council National Survey of Health and Development, a socially stratified sample of 5,362 singletons born in 1 week in March 1946 in England, Scotland, and Wales, of which 4,340 were analyzed.

**Predictor:**

Early-life overweight latent classes (never, prepubertal only, pubertal onset, or always), derived from repeated measurements of body mass index between ages 2 and 20 years.

**Outcomes & Measurements:**

The primary outcome was chronic kidney disease (CKD), defined as creatinine- or cystatin C–based estimated glomerular filtration rate (eGFR_cr_ and eGFR_cys_, respectively) <60 mL/min/1.73 m^2^ or urine albumin-creatinine ratio (UACR) ≥3.5 mg/mmol measured at age 60-64 years. Associations were explored through regression analysis, with adjustment for socioeconomic position, smoking, physical activity level, diabetes, hypertension, and overweight at ages 36 and 53 years.

**Results:**

2.3% of study participants had eGFR_cr_ <60 mL/min/1.73 m^2^, 1.7% had eGFR_cys_ <60 mL/min/1.73 m^2^, and 2.9% had UACR ≥3.5 mg/mmol. Relative to being in the never-overweight latent class, being in the pubertal-onset– or always-overweight latent classes was associated with eGFR_cys_-defined CKD (OR, 2.04; 95% CI, 1.09-3.82). Associations with CKD defined by eGFR_cr_ (OR, 1.27; 95% CI, 0.71-2.29) and UACR (OR, 1.33; 95% CI, 0.70-2.54) were less marked, but in the same direction. Adjustment for lifestyle and health factors had little impact on effect estimates.

**Limitations:**

A low prevalence of CKD resulted in low statistical power. No documentation of chronicity for outcomes. All-white study population restricts generalizability.

**Conclusions:**

Being overweight in early life was found to be associated with eGFR_cys_-defined CKD in later life. The associations with CKD defined by eGFR_cr_ and UACR were less marked, but in the same direction. Reducing or preventing overweight in the early years of life may significantly reduce the burden of CKD in the population.

During recent decades, the prevalence of overweight and obesity has increased dramatically in many parts of the world,^1^ with further increases predicted in the coming years.^2^ Of particular concern is the growing global childhood obesity epidemic.^3^

Globally increasing cardiovascular mortality^4^ together with the recognition of kidney disease as a cardiovascular risk factor^5^ has led to greater interest in the relationship between obesity and kidney disease. A growing number of studies have concluded that adulthood obesity increases the risk of kidney disease^6^ and numerous studies have tracked obesity from childhood into adulthood.^7^ However, not many studies have directly examined the effect of childhood obesity on the risk of kidney disease, and consequently, our understanding is limited. A review of 6 such studies determined that childhood obesity is associated with an elevated risk of kidney disease, as well as its progression and mortality,^8^ but all these studies were conducted in patients already with kidney disease rather than the general population. A more recent study of 1.2 million Israelis found overweight and obesity at age 17 years to be associated with a significantly increased risk of all-cause end-stage renal disease in a 25-year period.^9^

We previously have found overweight in adulthood, particularly overweight starting in early adulthood, to be associated with reduced kidney function later in life in the Medical Research Council (MRC) National Survey of Health and Development (NSHD).^10^ In the present study, we extend our previous work to explore whether the increase in risk associated with overweight stretches back even farther, into early life. We used repeated measurements of body mass index (BMI) between ages 2 and 20 years to model early-life overweight trajectories, which we then related to measures of chronic kidney disease (CKD) at age 60-64 years.

## Methods

### Participants

The NSHD is a socially stratified sample of 5,362 singletons born in 1 week in March 1946 in England, Scotland, and Wales who have been followed up many times since birth.^11^ Between October 2006 and February 2011 (when members of the cohort were aged 60-64 years), 2,856 eligible study participants (those known to be alive and with a known address in England, Scotland, or Wales) were invited for an assessment at 1 of 6 clinical research facilities. If they were unable or unwilling to come to one of the clinical research facilities, they were offered a slightly less comprehensive examination carried out in their own home by a trained nurse. Of those invited, 2,229 (78.0%) were assessed: 1,690 (59.2%) attended a clinic and 539 (18.9%) had a home visit.^12^

### Measures

Height and weight were measured at ages 2, 4, 6, 7, 11, and 15 according to standard protocols and self-reported at age 20 years. BMI, defined as weight in kilograms divided by height in meters squared, was calculated at each age. We then used the international overweight cutoffs of Cole et al^13^ to determine the corresponding overweight status at each age. For clarity, those identified as overweight by this approach include both those traditionally labeled overweight and those labeled obese. Furthermore, in this report, we use the term overweight as a noun, as is customary with obese.

At the clinic or home visit at age 60-64 years, blood and urine samples were obtained and processed according to standardized protocols. Serum creatinine was measured by means of a kinetic version of the Jaffé method using a Siemens Dimension Xpand analyzer at the MRC Human Nutrition Research laboratory in Cambridge. A method-specific correction, specified by the UK National External Quality Assessment Service, was applied to standardize measured values to the reference method (isotope-dilution mass spectrometry). Cystatin C was measured by an automated particle-enhanced immunoturbidimetric assay at the Department of Clinical Biochemistry at the Glasgow Royal Infirmary. Urine creatinine was measured using a kinetic version of the Jaffé method on a Siemens Dimension analyzer, and urinary albumin was measured by an immunoturbidimetric method on a Siemens BNII/ProSpec analyzer at the MRC Human Nutrition Research laboratory in Cambridge.

Creatinine- and cystatin C−based estimated glomerular filtration rate (eGFR_cr_ and eGFR_cys_, respectively) were calculated using the CKD Epidemiology Collaboration (CKD-EPI) formulas dating from 2009 and 2012, respectively.^14^, ^15^ Urine albumin-creatinine ratio (UACR) was calculated with adjustment for storage time.

Based on KDIGO (Kidney Disease: Improving Global Outcomes) criteria,^16^ we defined CKD as either: (1) eGFR_cr_ <60 mL/min/1.73 m^2^, (2) eGFR_cys_ <60 mL/min/1.73 m^2^, or (3) UACR ≥3.5 mg/mmol. We analyzed each of these CKD outcomes separately, as well as a composite measure that indicated whether CKD was present on any one or more of the CKD outcomes. As a secondary outcome, we also analyzed cystatin C.

Several lifestyle and health factors were considered as potential confounders or mediators of the association between overweight trajectory during ages 2-20 years and later CKD: childhood socioeconomic position (SEP; manual labor/blue collar work vs nonmanual labor/white collar work) was derived from the father's occupation when the study participant was aged 4 years; adulthood SEP (as described) was defined as the highest occupational class derived from the study participant's and spouse's occupations at age 53 years; previously derived smoking trajectory between ages 20 and 53 years^17^; previously derived physical activity latent classes between ages 31 and 53 years^18^; diabetes (self-reported physician-diagnosed diabetes by age 60-64 years, on diabetes medication at age 60-64 years, and hemoglobin A_1c_ level at age 60-64 years); and hypertension (previously derived systolic blood pressure latent trajectory between ages 36 and 53 years,^19^ on blood pressure medication at age 60-64 years, and measured systolic blood pressure at age 60-64 years).

Overweight at ages 36 and 53 years was considered as potential mediators. Height and weight, measured at ages 36 and 53 years using standard protocols, were used to calculate BMI, with overweight defined as BMI ≥25 kg/m^2^.

### Statistical Analyses

We first conducted longitudinal latent class analysis on the overweight indicator variables between ages 2 and 20 years. The objective of longitudinal latent class analysis is to decide how many latent classes are required to describe the data and categorize individuals into their most likely classes given their observed measurements. Analyses were restricted to study members with at least one BMI measurement between ages 2 and 20 years. Data missingness was handled using full information maximum likelihood under the assumption of missing at random.^20^

Patterns of early-life (ages 2-20 years) overweight were found to differ between males and females, so separate longitudinal latent class analyses were conducted. We used a variety of different tools to decide how many classes were required because no single approach is commonly accepted.^21^ Posterior probabilities were derived to quantify the probability with which an individual with a given early-life overweight pattern belonged to each latent class. We investigated to what extent the latent classes were associated with birth weight.

### Main Analyses

To account for the potential bias caused by missing data, we conducted our analyses using a multiple imputation approach.^22^, ^23^ In addition to all variables included in the analysis models, the imputation model also included occupation at other ages in adulthood, further repeated measures of adulthood BMI, repeated measures of adulthood waist to hip ratio, birth weight, achieved educational levels of the study member and their parents, and response at the age 60- to 64-year data collection (eg, clinic/home visit, temporary/permanent refusal, and untraced). Interactions with sex were included in the imputation model for all variables. Study members who were known to have died prior to or during the age 60- to 64-year data collection were excluded from the multiple imputation analysis. Fifty imputed data sets were obtained through chained equations.^24^, ^25^

Each of the 4 binary CKD outcomes was related to the resultant overweight latent classes using logistic regression, weighted by the longitudinal latent class analysis posterior class membership probabilities and minimally adjusted for sex and age at CKD measurements.

With the exception of childhood SEP, identifying lifestyle variables (SEP, smoking, and physical activity) as either potential confounders or mediators of the association between early-life overweight and later CKD is not straightforward. However, adulthood diabetes and hypertension more clearly are potential mediators of the association of interest. The extent to which these lifestyle and health factors confounded or mediated the association between early-life overweight and later CKD was examined by adding, in turn, childhood and adulthood SEP, lifetime smoking trajectory, midadulthood physical activity levels, diabetes (all 3 variables), hypertension (all 3 variables), and finally, all lifestyle and health factors to the minimally adjusted models.

The extent to which the association between early-life overweight and later CKD was mediated by adulthood overweight was examined by adding, in turn, overweight at age 36 years and overweight at age 53 years to the minimally adjusted models.

### Supplementary Analyses

Cystatin C level was related to the early-life overweight latent classes using linear regression, again weighted by the longitudinal latent class analysis posterior class membership probabilities and minimally adjusted for sex and age at the CKD measurements.

All analyses then were repeated using only complete cases for comparison with multiple imputation results.

No interactions between sex and early-life overweight latent class were found in any model, so combined male and female models are presented throughout.

The longitudinal latent class analysis was conducted using Mplus 6 (Muthén & Muthén), with the remaining analysis, including the multiple imputation, performed using Stata 12 (StataCorp).

## Results

### Study Participants and Longitudinal Latent Class Analysis

A flow diagram showing inclusion in the early-life (ages 2-20 years) overweight longitudinal latent class analysis and multiple imputation analysis is shown in Fig S1 (provided as online supplementary material). Of the original 5,362 study members in the cohort, 4,884 had at least one BMI measurement between ages 2 and 20 years and were included in the longitudinal latent class analysis. Of these, 77.6% had at least 5 of the 7 BMI measurements. The prevalence of early-life overweight decreased from 35.1% at age 2 years to 7.1% at age 7 years before increasing to 12.6% at age 20 years (Table S1).

**Figure 1:**
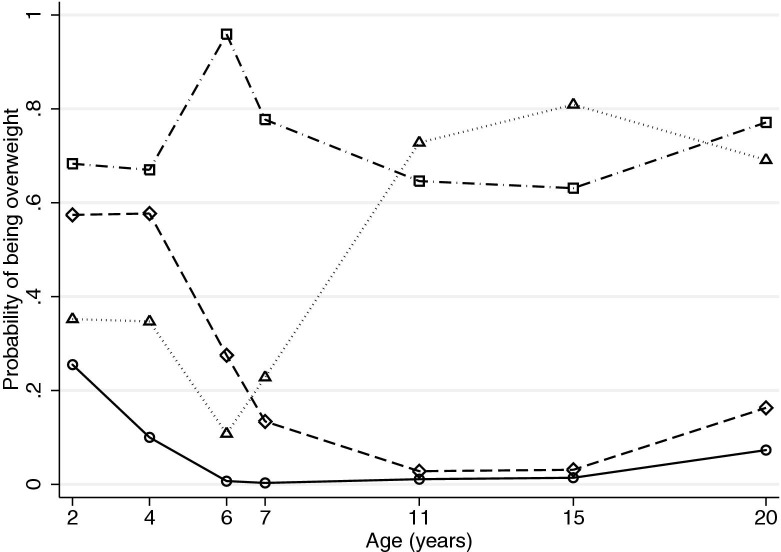
Early-life overweight latent class profiles for males (n = 2,564). Solid line and circles, never overweight (71.4%); dashed line and diamonds, prepubertal-only overweight (21.0%); dotted line and triangles, pubertal-onset overweight (5.0%); and dot-dashed line and squares, always overweight (2.7%).

**Figure 2:**
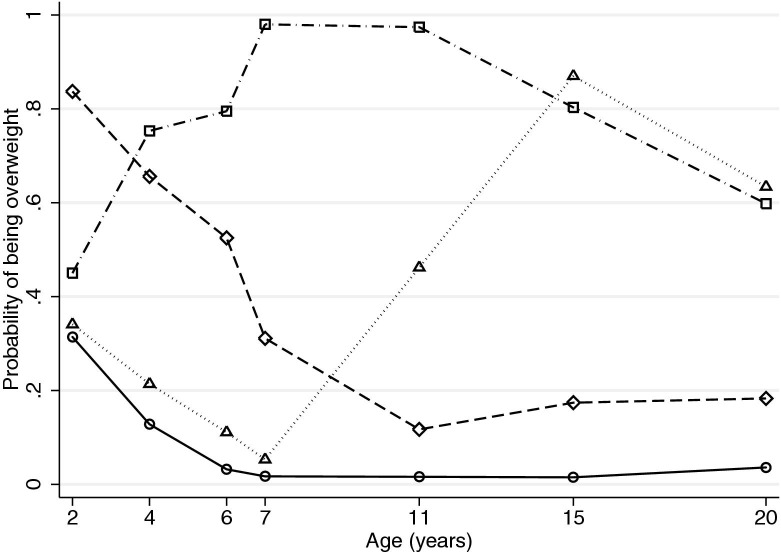
Early-life overweight latent class profiles for females (n = 2,320). Solid line and circles, never overweight (80.6%); dashed line and diamonds, prepubertal-only overweight (8.9%); dotted line and triangles, pubertal-onset overweight (6.6%); and dot-dashed line and squares, always overweight (3.9%).

In the longitudinal latent class analysis, all metrics suggested that 4 early-life overweight latent classes were required for both males and females (results not shown). These patterns of early-life overweight can be considered as never (71.4% of males and 80.6% of females), prepubertal only (21.0% and 8.9%), pubertal onset (5.0% and 6.6%), and always (2.7% and 3.9%; Figs 1 and 2). Males and females in the never-overweight latent class had the lowest average birth weight (3.44 and 3.30 kg, respectively), and those in the always-overweight class had the highest (3.68 and 3.49 kg), with birth weights in the prepubertal-only (3.60 and 3.47 kg) and pubertal-onset (3.61 and 3.39 kg) overweight classes falling in between.

The observed overall prevalence of CKD varied between the different measures, from 1.7% (eGFR_cys_) to 2.9% (UACR). Median cystatin C level was 0.81 (interquartile range, 0.16) mg/L. Overall distributions of all variables included in the analysis are listed in Table 1.

**Table 1:** Distributions of Variables Stratified by Early-Life Overweight Latent Class, Weighted by Posterior Probability of Class Membership

	No. With Available Data	Total (N = 4,884^a^)	Early-Life Overweight			
			Never (n = 4,037)	Prepubertal Only (n = 491)	Pubertal Onset (n = 209)	Always (n = 147)
Age at examination^b^ (y)	2,197	63.6 [1.3]	63.6 [1.3]	63.7 [1.5]	63.7 [1.6]	63.6 [1.3]
eGFR_cr_ at age 60-64 y (mL/min/1.73 m^2^)	1,826	96.0 [16.3]	96.3 [16.1]	95.5 [16.4]	94.6 [16.2]	96.8 [18.5]
eGFR_cys_ at age 60-64 y (mL/min/1.73 m^2^)	2,022	96.9 [19.0]	96.9 [18.8]	98.3 [18.9]	92.1 [20.5]	96.4 [19.1]
UACR at age 60-64 y (mg/mmol)	2,141	0.55 [0.44]	0.54 [0.43]	0.54 [0.40]	0.61 [0.50]	0.62 [0.53]
Cystatin C at age 60-64 y (mg/L)	2,023	0.81 [0.16]	0.81 [0.16]	0.81 [0.16]	0.84 [0.18]	0.82 [0.14]
HbA_1c_ at age 60-64 y (%)	2,014	5.7 [0.5]	5.7 [0.5]	5.7 [0.4]	5.7 [0.6]	5.7 [0.5]
SBP at age 60-64 y (mm Hg)	2,177	133 [22]	132 [22]	133 [22]	135 [23]	134 [21]
eGFR_cr_ <60 mL/min/1.73 m^2^ at age 60-64 y	1,826	2.3	2.0	2.3	4.6	2.9
eGFR_cys_ <60 mL/min/1.73 m^2^ at age 60-64 y	2,022	1.7	1.4	1.7	5.3	3.2
UACR ≥3.5 mg/mmol at age 60-64 y	2,141	2.9	2.9	2.5	4.5	1.8
CKD by composite measure at age 60-64 y	1,799	5.5	5.1	5.0	11.9	5.8
Manual labor/blue collar work						
Study member's parents	4,473	59.2	58.9	59.8	63.9	56.4
Study member at age 53 y	2,828	21.0	20.4	23.6	25.6	15.3
Lifetime smoking trajectory	3,352					
Never smoker		28.0	28.6	25.8	27.5	26.5
Predominantly nonsmoker		32.6	32.7	32.4	30.2	36.5
Predominantly smoker		21.0	20.9	21.6	19.9	24.7
Lifelong smoker		18.3	17.8	20.3	22.5	12.3
Time sitting down during day at age 36 y	3,238					
Much		26.4	26.0	27.5	27.8	27.5
Average		34.3	34.5	34.9	30.1	35.3
Little		39.3	39.5	37.7	42.2	37.2
Low walking latent class at age 36-43 y	3,522	41.8	41.1	44.6	43.9	42.9
Low cycling latent class at age 31-43 y	3,711	87.4	87.4	86.9	87.7	89.8
Leisure time physical activity latent class at age 36-53 y	3,601					
Low		46.8	46.4	46.7	53.3	44.9
Gardening and DIY		18.2	17.5	20.2	19.9	20.8
Sport and leisure		35.0	36.1	33.1	26.9	34.3
Self-reported diabetes by age 60-64 y	2,417	7.5	6.8	7.4	12.8	14.0
Receiving diabetes treatment at age 60-64 y	2,585	5.5	5.0	5.6	7.6	13.1
Increased/high midlife SBP trajectory	3,589	6.5	6.7	4.9	6.6	8.9
Receiving antihypertensive treatment at age 60-64 y	2,585	29.7	29.2	28.4	35.1	36.3
Overweight at age 36 y	3,233	35.0	28.9	44.0	68.4	75.4
Overweight at age 53 y	2,895	66.8	63.2	73.2	85.8	87.7

*Note:* With the exception of study members being given as number, values in upper section of table are given as median [interquartile range] and values in lower section are given as percentage.

Abbreviations: CKD, chronic kidney disease; DIY, do-it-yourself activities; eGFR_cr_, creatinine-based estimated glomerular filtration rate; eGFR_cys_, cystatin C−based estimated glomerular filtration rate; HbA_1c_, hemoglobin A_1c_; SBP, systolic blood pressure; UACR, urinary albumin-creatinine ratio.

a Of original 5,362 study members in cohort, 4,884 had one or more body mass index measurement between ages 2 and 20 years and were included in longitudinal latent class analysis.

b The examination in question occurred between October 2006 and February 2011, when cohort members were aged 60-64 years.

The prevalence of CKD differed between the early-life BMI latent classes (Table 1). For both eGFR_cr_ and eGFR_cys_, the lowest prevalence was in the never-overweight latent class (2.0% and 1.4%, respectively), with the highest prevalence in the pubertal-onset overweight latent class (4.6% and 5.3%). For UACR, the highest prevalence similarly was in the pubertal-onset overweight latent class (4.5%), although the lowest prevalence was in the always-overweight latent class (1.8%). Distributions of all variables included in the analysis within each early-life BMI latent class also are listed in Table 1.

There were very few study participants in the always-overweight latent class with CKD, so for the purpose of the remaining analyses, the pubertal-onset and always-overweight latent classes were combined. However, although there was insufficient power to formally test it, there was a suggestion that CKD risk was greater in the pubertal-onset overweight latent class than in the always-overweight latent class.

### Main Analyses

As shown in Fig S1, 4,340 study members were eligible for both the longitudinal latent class analysis and multiple imputation procedures and were included in the analysis. There was evidence that, relative to being in the never-overweight latent class, being in the pubertal-onset or always-overweight latent classes was associated with CKD defined using eGFR_cys_ (odds ratio [OR], 2.04; 95% confidence interval [CI], 1.09-3.82; Table 2, model 1). The associations when considering eGFR_cr_ (OR, 1.27; 95% CI, 0.71-2.29) and UACR (OR, 1.33; 95% CI, 0.70-2.54) were less marked, but in the same direction. There also was evidence of an association with the composite CKD measure (OR, 1.51; 95% CI, 1.03-2.20). For none of the outcomes was there evidence that being in the prepubertal-only overweight latent class increased the risk of reduced kidney function relative to the never-overweight latent class.

**Table 2:** ORs for CKD at Age 60-64 Years by Early-Life Overweight Latent Class, Models 1-3

CKD Definition and Early-Life Overweight Latent Class	Percentage in Latent Class		Model 1^a^		Model 2^b^		Model 3^c^	
	Total^d^	With CKD^d^	OR (95% CI)	*P*	OR (95% CI)	*P*	OR (95% CI)	*P*
CKD by eGFR_cr_								
Never	76.0	3.5	1.00 (reference)	—	1.00 (reference)	—	1.00 (reference)	—
Prepubertal only	15.1	3.6	1.00 (0.66-1.53)	0.9	1.00 (0.66-1.52)	0.9	1.00 (0.66-1.53)	0.9
Pubertal onset/always	8.9	4.5	1.27 (0.71-2.29)	0.4	1.26 (0.70-2.27)	0.4	1.27 (0.70-2.29)	0.4
CKD by eGFR_cys_								
Never	76.0	2.0	1.00 (reference)	—	1.00 (reference)	—	1.00 (reference)	—
Prepubertal only	15.1	2.1	1.21 (0.70-2.11)	0.5	1.20 (0.69-2.07)	0.5	1.19 (0.68-2.08)	0.5
Pubertal onset/always	8.9	4.1	2.04 (1.09-3.82)	0.03	1.98 (1.06-3.70)	0.03	2.00 (1.06-3.79)	0.03
CKD by UACR								
Never	76.0	2.7	1.00 (reference)	—	1.00 (reference)	—	1.00 (reference)	—
Prepubertal only	15.1	2.8	0.93 (0.60-1.42)	0.7	0.93 (0.60-1.42)	0.7	0.92 (0.60-1.41)	0.7
Pubertal onset/always	8.9	3.6	1.33 (0.70-2.54)	0.4	1.32 (0.69-2.53)	0.4	1.32 (0.69-2.52)	0.4
Composite CKD measure								
Never	76.0	7.3	1.00 (reference)	—	1.00 (reference)	—	1.00 (reference)	—
Prepubertal only	15.1	7.4	0.99 (0.74-1.34)	0.9	0.99 (0.74-1.33)	0.9	0.99 (0.74-1.33)	0.9
Pubertal onset/always	8.9	10.7	1.51 (1.03-2.20)	0.03	1.49 (1.02-2.17)	0.04	1.50 (1.02-2.19)	0.04

*Note:* Multiple imputation analysis (n = 4,340).

Abbreviations and definitions: CI, confidence interval; CKD, chronic kidney disease (eGFR_cr_ or eGFR_cys_ <60 mL/min/1.73 m^2^ or UACR ≥3.5 mg/mmol); eGFR_cr_, creatinine-based estimated glomerular filtration rate; eGFR_cys_, cystatin C–based estimated glomerular filtration rate; OR, odds ratio; UACR, urinary albumin-creatinine ratio.

a Model 1: adjusted for sex and age at CKD measurements.

b Model 2: adjusted for sex, age at CKD measurements, and childhood and adulthood socioeconomic position.

c Model 3: adjusted for sex, age at CKD measurements, and lifetime smoking trajectory.

d Average across all 50 imputed data sets.

Adjustment for lifestyle and health factors had little impact on effect estimates (Tables 2 and 3). Adjustment for diabetes and hypertension generally resulted in the greatest attenuation. In the fully adjusted models (Table 3, model 7), there remained some evidence of an association with CKD defined using eGFR_cys_ (OR, 1.88; 95% CI, 0.99-3.58) or the composite measure (OR, 1.39; 95% CI, 0.94-2.04).

**Table 3:** ORs for CKD at Age 60-64 Years by Early-Life Overweight Latent Class, Models 4-7

CKD Definition and Early-Life Overweight Latent Class	Model 4^a^		Model 5^b^		Model 6^c^		Model 7^d^	
	OR (95% CI)	*P*	OR (95% CI)	*P*	OR (95% CI)	*P*	OR (95% CI)	*P*
CKD by eGFR_cr_								
Never	1.00 (reference)	—	1.00 (reference)	—	1.00 (reference)	—	1.00 (reference)	—
Prepubertal only	1.00 (0.66-1.53)	0.9	1.01 (0.67-1.54)	0.9	1.02 (0.67-1.55)	0.9	1.02 (0.67-1.56)	0.9
Pubertal onset/always	1.27 (0.70-2.29)	0.4	1.23 (0.68-2.23)	0.5	1.23 (0.68-2.23)	0.5	1.20 (0.66-2.19)	0.5
CKD by eGFR_cys_								
Never	1.00 (reference)	—	1.00 (reference)	—	1.00 (reference)	—	1.00 (reference)	—
Prepubertal only	1.22 (0.70-2.12)	0.5	1.23 (0.70-2.14)	0.5	1.23 (0.71-2.13)	0.5	1.20 (0.69-2.12)	0.5
Pubertal onset/always	2.04 (1.08-3.85)	0.03	1.97 (1.05-3.69)	0.03	1.96 (1.04-3.67)	0.04	1.88 (0.99-3.58)	0.06
CKD by UACR								
Never	1.00 (reference)	—	1.00 (reference)	—	1.00 (reference)	—	1.00 (reference)	—
Prepubertal only	0.93 (0.60-1.42)	0.7	0.93 (0.61-1.43)	0.7	0.94 (0.61-1.45)	0.8	0.94 (0.61-1.45)	0.8
Pubertal onset/always	1.29 (0.67-2.47)	0.4	1.26 (0.66-2.41)	0.5	1.24 (0.65-2.39)	0.5	1.18 (0.61-2.27)	0.6
Composite CKD measure								
Never	1.00 (reference)	—	1.00 (reference)	—	1.00 (reference)	—	1.00 (reference)	—
Prepubertal only	1.00 (0.74-1.34)	0.9	1.00 (0.75-1.35)	0.9	1.01 (0.75-1.36)	0.9	1.01 (0.75-1.36)	0.9
Pubertal onset/always	1.48 (1.02-2.17)	0.04	1.45 (0.99-2.13)	0.06	1.44 (0.98-2.11)	0.06	1.39 (0.94-2.04)	0.1

*Note:* Multiple imputation analysis (n = 4,340).

Abbreviations and definitions: CI, confidence interval; CKD, chronic kidney disease (defined as eGFR_cr_ or eGFR_cys_ <60 mL/min/1.73 m^2^ or urine ACR ≥3.5 mg/mmol); eGFR_cr_, creatinine-based estimated glomerular filtration rate; eGFR_cys_, cystatin C–based estimated glomerular filtration rate; OR, odds ratio; UACR, urinary albumin-creatinine ratio.

a Model 4: adjusted for sex, age at CKD measurements, and midadulthood physical activity trajectories.

b Model 5: adjusted for sex, age at CKD measurements, and diabetes.

c Model 6: adjusted for sex, age at CKD measurements, and hypertension.

d Model 7: adjusted for sex, age at CKD measurements, childhood and adulthood socioeconomic position, lifetime smoking trajectory, midadulthood physical activity trajectories, diabetes, and hypertension.

All effect estimates showed greater attenuation when adjusted for overweight at age 36 years than when adjusted for overweight at age 53 years (Table 4). For example, the composite CKD measure OR for pubertal onset or always overweight was attenuated from 1.51 to 1.18 on adjustment for overweight at age 36 years, but to only 1.39 on adjustment for overweight at age 53 years.

**Table 4:** ORs for CKD at Age 60-64 Years by Early-Life Overweight Latent Class, Adjusted for Later-Life Overweight

CKD Definition and Early-Life Overweight Latent Class	Model 1^a^		Model 1 + Overweight at Age 36 y^b^		Model 1 + Overweight at Age 53 y^c^	
	OR (95% CI)	*P*	OR (95% CI)	*P*	OR (95% CI)	*P*
CKD by eGFR_cr_						
Never	1.00 (reference)	—	1.00 (reference)	—	1.00 (reference)	—
Prepubertal only	1.00 (0.66-1.53)	0.9	0.94 (0.61-1.43)	0.8	0.98 (0.64-1.48)	0.9
Pubertal onset/always	1.27 (0.71-2.29)	0.4	1.01 (0.54-1.86)	0.9	1.16 (0.64-2.12)	0.6
CKD by eGFR_cys_						
Never	1.00 (reference)	—	1.00 (reference)	—	1.00 (reference)	—
Prepubertal only	1.21 (0.70-2.11)	0.5	1.11 (0.64-1.92)	0.7	1.16 (0.67-2.02)	0.6
Pubertal onset/always	2.04 (1.09-3.82)	0.03	1.54 (0.82-2.86)	0.2	1.80 (0.95-3.38)	0.07
CKD by UACR						
Never	1.00 (reference)	—	1.00 (reference)	—	1.00 (reference)	—
Prepubertal only	0.93 (0.60-1.42)	0.7	0.87 (0.57-1.34)	0.5	0.91 (0.59-1.40)	0.7
Pubertal onset/always	1.33 (0.70-2.54)	0.4	1.09 (0.57-2.06)	0.8	1.25 (0.66-2.39)	0.5
Composite CKD measure						
Never	1.00 (reference)	—	1.00 (reference)	—	1.00 (reference)	—
Prepubertal only	0.99 (0.74-1.34)	0.9	0.93 (0.69-1.24)	0.6	0.97 (0.72-1.30)	0.8
Pubertal onset/always	1.51 (1.03-2.20)	0.03	1.18 (0.80-1.75)	0.4	1.39 (0.95-2.04)	0.09

*Note:* Multiple imputation analysis (n = 4,340).

Abbreviations and definitions: CI, confidence interval; CKD, chronic kidney disease (defined as eGFR_cr_ or eGFR_cys_ <60 mL/min/1.73 m^2^ or urine ACR ≥3.5 mg/mmol); eGFR_cr_, creatinine-based estimated glomerular filtration rate; eGFR_cys_, cystatin C–based estimated glomerular filtration rate; OR, odds ratio; UACR, urinary albumin-creatinine ratio.

a Model 1: adjusted for sex and age at CKD measurements (data repeated from Table 2).

b Model 2: adjusted for sex, age at CKD measurements, and overweight at age 36 years.

c Model 3: adjusted for sex, age at CKD measurements, and overweight at age 53 years.

### Supplementary Analyses

Being in the pubertal-onset or always-overweight latent classes also was associated with a 0.027 (95% CI, 0.007-0.048) mg/L increase in cystatin C level relative to being in the never-overweight latent class (Table S2*a*). Adjustment for lifestyle and health factors again had little impact on this effect estimate (Table S2*a* and *b*), and it showed greater attenuation when adjusted for overweight at age 36 years than when adjusted for overweight at age 53 years (Table S2*c*).

Complete case analyses of the binary CKD outcomes included between 1,799 (composite CKD measure) and 2,141 (UACR) study members. Estimated associations between early-life overweight latent class and later CKD were stronger than those in the multiple imputation analysis for eGFR_cr_ and eGFR_cys_, but similar for UACR (Table S3*a*). However, the CIs were much wider due to the reduced sample size. There remained strong evidence of associations for eGFR_cys_ and the composite CKD measure, with associations for eGFR_cr_ and UACR less marked but in the same direction. Additional adjustment for lifestyle factors again resulted in little attenuation of the effect estimates (Table S3*b* and *c*). As in the multiple imputation analysis, all effect estimates showed greater attenuation when adjusted for overweight at age 36 years than when adjusted for overweight at age 53 years (Table S3*d*).

Complete case results for cystatin C were in line similarly with the multiple imputation results (Table S4*a-d*).

## Discussion

In a large population-based prospective study, we found that overweight throughout early life or becoming overweight in the period from puberty to age 20 years was associated with CKD at age 60-64 years assessed using eGFR_cys_. This association was supported by less marked associations with eGFR_cr_ and UACR. Confounding or mediation by childhood and adulthood SEP, lifetime smoking trajectory, and midadulthood physical activity levels did not explain the observed associations, and mediation by diabetes and hypertension was limited.

The associations were attenuated to a far greater extent when adjusted for overweight at age 36 years than when adjusted for overweight at age 53 years. This suggests that: (1) being overweight in early adulthood is particularly harmful for later CKD (corresponding to our previous findings in this cohort^10^), and/or (2) early-life and late-adulthood overweight have independent effects on CKD risk. However, this finding also may be due in part to the stronger association of early-life overweight with overweight at age 36 years than overweight at age 53 years.

To our knowledge, there are no existing studies of the effect of overweight throughout early life on later kidney disease in general population cohorts. In a review of studies examining the association between obesity and kidney disease, Wang et al^8^ identified 6 cohort studies in youth, all of which involved participants who were either kidney disease patients or transplant recipients. In addition, the variety of outcomes and relatively short follow-up periods make direct comparison with the present study difficult. Wang et al^8^ concluded that high BMI or obesity in early life is associated with an increased risk of kidney disease. This finding is certainly not at odds with ours.

In a recent large population-based cohort study, Vivante et al^9^ found overweight and obesity at age 17 years to be associated with significantly increased risk of all-cause end-stage renal disease in a 25-year period. Although their adjusted hazard ratios of 3.00 (95% CI, 2.50-3.60) for overweight and 6.89 (95% CI, 5.52-8.59) for obesity appear somewhat stronger than the ORs reported in the present study, differences in definition of both the exposure and outcome make comparison difficult.

Associations between early-life overweight and many adverse health outcomes in later life are now acknowledged,^26^ and several previous studies have looked in more detail at specific patterns of early-life weight gain. In the same cohort as the present study, Hardy et al^27^ highlighted excessive pubertal BMI gain as being associated particularly strongly with later blood pressure. Our observation that the pubertal-onset overweight latent class seemed at somewhat higher risk of later CKD than the always-overweight latent class hints at a similar pattern for CKD.

Previous studies also have found children who were overweight before puberty but returned to normal weight during puberty to have similar levels of later blood pressure^28^ and other cardiovascular risk factors^29^ as those who remained normal weight throughout. Our finding that CKD risk did not differ between the prepubertal-only and never-overweight latent classes is in agreement with these previous results.

Studies also have suggested that individuals who were born small but then rapidly gained weight during early life are at particularly high risk of later hypertension^30^, ^31^ or diabetes.^31^ However, the pubertal-onset overweight latent class identified in the present analysis was found to have a relatively high mean birth weight in both males and females, so our results cannot be explained by this pathway.

The mechanisms relating early-life BMI to later CKD are not well researched. Overweight children are at higher risk of developing high blood pressure and diabetes, although evidence for effects independent of adulthood overweight is lacking.^26^ Overweight may have independent associations with blood pressure that may precede decreased kidney function.^32^ Animal studies suggest that obesity is followed by initial glomerular hyperfiltration, the development of albuminuria, and subsequent decline in eGFR.^33^, ^34^ This may explain why we did not observe strong associations of early-life overweight with UACR; one could speculate that at age 60-64 years, those who were overweight from early in life may well have passed through the early albuminuria stage, although the data do not allow the sequence of events to be disentangled.

There are many strengths to this analysis. BMI measurements were available at regular intervals throughout early life and calculating overweight status using age-specific cutoffs allowed the variability in actual ages at measurement to be taken into account. Furthermore, the availability of several different measures of CKD allowed a more thorough analysis.

The use of full information maximum likelihood in the longitudinal latent class analysis allowed the inclusion of all study members with at least one early-life BMI measurement under the assumption of missing at random.^20^ The longitudinal latent class analysis models fit the data well and the resultant latent classes were clearly separated and easily interpreted.

Cohort members remaining in the NSHD at the time of data collection at age 53 years^35^ and at age 60-64 years^12^ have been determined as generally representative of native-born adults living in England, Scotland, and Wales. We thus are confident that our analysis sample retained the representativeness of the study population as a whole. Use of multiple imputation allowed us to account for the potential bias caused by missing data.

There also were limitations to the study. Calculation of eGFR, rather than measuring GFR directly, may have resulted in some misclassification of CKD. The lack of repeated measurements of kidney function meant that we were unable to ascertain CKD using the recommended definition of reduced kidney function for at least 3 months.^16^ This is likely to introduce nondifferential misclassification, meaning that the true association is even stronger than described here. In addition, the low prevalence of CKD in this cohort resulted in low statistical power. Although the similarity in findings across the different measures of CKD and cystatin C suggests robustness to our findings, it is important that they are replicated in larger general population cohorts. Finally, although we had good coverage of different UK regions and social class groupings, because the NSHD study population is all white, our findings cannot necessarily be extrapolated to the nonwhite British population.

It should be noted that members of this cohort experienced childhood in the 1950s, when overweight/obesity was far less prevalent. Only 7% of study members were in the pubertal-onset or always-overweight latent classes, whereas ∼40% of modern children in the Americas and Europe are thought to be overweight or obese.^3^ Thus, the absolute impact of any effect of early-life overweight on CKD is likely to be substantial at the population level.

In conclusion, being overweight throughout early life or becoming overweight in the period from puberty to age 20 years was found to be associated with CKD in later life. Reducing or preventing overweight in early life may significantly reduce the burden of CKD in the population.

## Supplementary Material

Supplementary Table S1 (PDF) Prevalence of overweight at each age in early life in the MRC NSHD.

Supplementary Table S2 (PDF) Linear regression coefficients for cystatin C level at age 60-64 by early-life overweight latent class.

Supplementary Table S3 (PDF) ORs for CKD at age 60-64 by early-life overweight latent class, by complete case analyses.

Supplementary Table S4 (PDF) Linear regression coefficients for cystatin C level at age 60-64 by early-life overweight latent class, by complete case analyses.

Supplementary Figure S1 (PDF) Flow diagram showing inclusion in early-life overweight latent class analysis and multiple imputation analysis.

## References

[1] World Health Organisation (2000).

[2] Kelly T., Yang W., Chen C.S., Reynolds K., He J. (2008). Global burden of obesity in 2005 and projections to 2030. Int J Obes.

[3] Wang Y., Lobstein T. (2006). Worldwide trends in childhood overweight and obesity. Int J Pediatr Obes.

[4] World Health Organisation (2008).

[5] Chronic Kidney Disease Prognosis Consortium (2010). Association of estimated glomerular filtration rate and albuminuria with all-cause and cardiovascular mortality in general population cohorts: a collaborative meta-analysis. Lancet.

[6] Wang Y., Chen X., Song Y., Caballero B., Cheskin L.J. (2008). Association between obesity and kidney disease: a systematic review and meta-analysis. Kidney Int.

[7] Singh A.S., Mulder C., Twisk J.W., van Mechelen W., Chinapaw M.J. (2008). Tracking of childhood overweight into adulthood: a systematic review of the literature. Obes Rev.

[8] Wang Y., Chen X., Klag M.J., Caballero B. (2006). Epidemic of childhood obesity: implications for kidney disease. Adv Chronic Kidney Dis.

[9] Vivante A., Golan E., Tzur D. (2012). Body mass index in 1.2 million adolescents and risk for end-stage renal disease. Arch Intern Med.

[10] Silverwood R.J., Pierce M., Thomas C. (2013). Association between younger age when first overweight and increased risk for CKD. J Am Soc Nephrol.

[11] Kuh D., Pierce M., Adams J. (2011). Cohort profile: updating the cohort profile for the MRC National Survey of Health and Development: a new clinic-based data collection for ageing research. Int J Epidemiol.

[12] Stafford M., Black S., Shah I. (2013). Using a birth cohort to study ageing: representativeness and response rates in the National Survey of Health and Development. Eur J Ageing.

[13] Cole T.J., Bellizzi M.C., Flegal K.M., Dietz W.H. (2000). Establishing a standard definition for child overweight and obesity worldwide: international survey. BMJ.

[14] Levey A.S., Stevens L.A., Schmid C.H. (2009). A new equation to estimate glomerular filtration rate. Ann Intern Med.

[15] Inker L.A., Schmid C.H., Tighiouart H. (2012). Estimating glomerular filtration rate from serum creatinine and cystatin C. N Engl J Med.

[16] Kidney Disease: Improving Global Outcomes (2013). KDIGO Clinical Practice Guideline for the Evaluation and Management of Chronic Kidney Disease. Kidney Int Suppl.

[17] Clennell S., Kuh D., Guralnik J.M., Patel K.V., Mishra G.D. (2008). Characterisation of smoking behaviour across the life course and its impact on decline in lung function and all-cause mortality: evidence from a British birth cohort. J Epidemiol Community Health.

[18] Silverwood R.J., Nitsch D., Pierce M., Kuh D., Mishra G.D. (2011). Characterizing longitudinal patterns of physical activity in mid-adulthood using latent class analysis: results from a prospective cohort study. Am J Epidemiol.

[19] Wills A.K., Lawlor D.A., Muniz-Terrera G. (2012). Population heterogeneity in trajectories of midlife blood pressure. Epidemiology.

[20] Little R.J.A., Rubin D.B. (2002).

[21] Nylund K.L., Asparouhov T., Muthen B.O. (2007). Deciding on the number of classes in latent class analysis and growth mixture modeling: a Monte Carlo simulation study. Struct Equ Modeling.

[22] Sterne J.A., White I.R., Carlin J.B. (2009). Multiple imputation for missing data in epidemiological and clinical research: potential and pitfalls. BMJ.

[23] Kenward M.G., Carpenter J. (2007). Multiple imputation: current perspectives. Stat Methods Med Res.

[24] van Buuren S., Boshuizen H.C., Knook D.L. (1999). Multiple imputation of missing blood pressure covariates in survival analysis. Stat Med.

[25] Royston P. (2009). Multiple imputation of missing values: further update of ice, with an emphasis on categorical variables. Stata J.

[26] Park M.H., Falconer C., Viner R.M., Kinra S. (2012). The impact of childhood obesity on morbidity and mortality in adulthood: a systematic review. Obes Rev.

[27] Hardy R., Wadsworth M.E., Langenberg C., Kuh D. (2004). Birthweight, childhood growth, and blood pressure at 43 years in a British birth cohort. Int J Epidemiol.

[28] Mamun A.A., Lawlor D.A., O'Callaghan M.J., Williams G.M., Najman J.M. (2005). Effect of body mass index changes between ages 5 and 14 on blood pressure at age 14: findings from a birth cohort study. Hypertension.

[29] Lawlor D.A., Benfield L., Logue J. (2010). Association between general and central adiposity in childhood, and change in these, with cardiovascular risk factors in adolescence: prospective cohort study. BMJ.

[30] Huxley R.R., Shiell A.W., Law C.M. (2000). The role of size at birth and postnatal catch-up growth in determining systolic blood pressure: a systematic review of the literature. J Hypertens.

[31] Barker D.J., Osmond C., Kajantie E., Eriksson J.G. (2009). Growth and chronic disease: findings in the Helsinki Birth Cohort. Ann Hum Biol.

[32] Silventoinen K., Magnusson P.K., Neovius M. (2008). Does obesity modify the effect of blood pressure on the risk of cardiovascular disease? A population-based cohort study of more than one million Swedish men. Circulation.

[33] Henegar J.R., Bigler S.A., Henegar L.K., Tyagi S.C., Hall J.E. (2001). Functional and structural changes in the kidney in the early stages of obesity. J Am Soc Nephrol.

[34] Wadsworth M.E., Butterworth S.L., Hardy R.J. (2003). The life course prospective design: an example of benefits and problems associated with study longevity. Soc Sci Med.

[35] Wolf G., Chen S., Han D.C., Ziyadeh F.N. (2002). Leptin and renal disease. Am J Kidney Dis.

