# Supplementary material for: Early-Life Overweight Trajectory and CKD in the 1946 British Birth Cohort Study
Source: Am J Kidney Dis. 2013 Aug;62(2):276–84. doi: 10.1053/j.ajkd.2013.03.032 (PMC3719096; doi:10.1053/j.ajkd.2013.03.032)
Supplement: Supplementary Table S1 (PDF) — Prevalence of overweight at each age in early life in the MRC NSHD. [file mmc1.pdf]

Table S1. Prevalence of overweight at each age in early life in the Medical Research Council National Survey of Health and Development.

Age (years)	Males		Females		Total	
	n/N	%	n/N	%	n/N	%
2	690/2046	33.7	659/1794	36.7	1349/3840	35.1
4	497/2198	22.6	408/1986	20.5	905/4184	21.6
6	192/2050	9.4	208/1841	11.3	400/3891	10.3
7	126/2057	6.1	157/1920	8.2	283/3977	7.1
11	134/2050	6.5	175/1887	9.3	309/3937	7.8
15	130/1881	6.9	201/1700	11.8	331/3581	9.2
20	256/1829	14.0	192/1735	11.1	448/3564	12.6
