# Supplementary material for: Early-Life Overweight Trajectory and CKD in the 1946 British Birth Cohort Study
Source: Am J Kidney Dis. 2013 Aug;62(2):276–84. doi: 10.1053/j.ajkd.2013.03.032 (PMC3719096; doi:10.1053/j.ajkd.2013.03.032)
Supplement: Supplementary Table S2 (PDF) — Linear regression coefficients for cystatin C level at age 60-64 by early-life overweight latent class. [file mmc2.pdf]

Table S2. Linear regression coefficients (coeff) for cystatin C at age 60-64 years by early-life overweight latent class. Multiple imputation analysis (n = 4340).

Table S2a.

Childhood overweight latent class	% in this latent class[A]	Mean (SD) cystatin C (mg/l)[A]	Model 1			Model 2			Model 3		
			Coeff	95 % CI	*P*	Coeff	95 % CI	*P*	Coeff	95 % CI	*P*
Cystatin C (mg/l)											
Never	76.0	0.823 (0.149)	(ref)			(ref)			(ref)		
Pre-pubertal only	15.1	0.827 (0.152)	0.000	-0.015, 0.014	0.9	-0.001	-0.015, 0.013	0.9	-0.001	-0.015, 0.014	0.9
Pubertal onset/always	8.9	0.851 (0.177)	0.027	0.007, 0.048	0.009	0.026	0.006, 0.047	0.01	0.027	0.006, 0.048	0.01

[A]Average across all 50 imputed datasets.
Model 1: Adjusted for sex and age at CKD measurements.
Model 2: Adjusted for sex, age at CKD measurements and childhood and adulthood socioeconomic position.
Model 3: Adjusted for sex, age at CKD measurements and lifetime smoking trajectory.

Table S2b.

Childhood overweight latent class	Model 4			Model 5			Model 6			Model 7		
	Coeff	95 % CI	*P*	Coeff	95 % CI	*P*	Coeff	95 % CI	*P*	Coeff	95 % CI	*P*
Cystatin C (mg/l)												
Never	(ref)			(ref)			(ref)			(ref)		
Pre-pubertal only	0.000	-0.014, 0.014	0.9	0.000	-0.014, 0.014	0.9	0.000	-0.014, 0.014	0.9	0.000	-0.014, 0.014	0.9
Pubertal onset/always	0.027	0.006, 0.048	0.01	0.027	0.006, 0.047	0.01	0.026	0.005, 0.047	0.01	0.025	0.005, 0.046	0.02

Model 4: Adjusted for sex, age at CKD measurements and mid-adulthood physical activity trajectories.
Model 5: Adjusted for sex, age at CKD measurements and diabetes.
Model 6: Adjusted for sex, age at CKD measurements and hypertension.
Model 7: Adjusted for sex, age at CKD measurements, childhood and adulthood socioeconomic position, lifetime smoking trajectory, mid-adulthood physical activity trajectories, diabetes and hypertension.

Table S2c.

Childhood overweight latent class	Model 1			Model 2			Model 3		
	Coeff	95 % CI	*P*	Coeff	95 % CI	*P*	Coeff	95 % CI	*P*
Cystatin C (mg/l)									
Never	(ref)			(ref)			(ref)		
Pre-pubertal only	0.000	-0.015, 0.014	0.9	-0.004	-0.018, 0.011	0.6	-0.002	-0.016, 0.012	0.8
Pubertal onset/always	0.027	0.007, 0.048	0.009	0.016	-0.005, 0.037	0.1	0.022	0.001, 0.042	0.04

Model 1: Adjusted for sex and age at CKD measurements.
Model 2: Adjusted for sex, age at CKD measurements and overweight at age 36 years.
Model 3: Adjusted for sex, age at CKD measurements and overweight at age 53 years.
