# Supplementary material for: Early-Life Overweight Trajectory and CKD in the 1946 British Birth Cohort Study
Source: Am J Kidney Dis. 2013 Aug;62(2):276–84. doi: 10.1053/j.ajkd.2013.03.032 (PMC3719096; doi:10.1053/j.ajkd.2013.03.032)
Supplement: Supplementary Table S3 (PDF) — ORs for CKD at age 60-64 by early-life overweight latent class, by complete case analyses. [file mmc3.pdf]

**Table S3. Odds ratios (OR) for chronic kidney disease at age 60-64 years by early-life overweight latent class.**

**Table S3a.**

| Childhood overweight latent class | n (%) in this latent class | n (%) of those in this latent class with CKD | OR | 95 % CI | *P* |
|---|---|---|---|---|---|
| Creatinine-based eGFR (n = 1826) | | | | | |
| Never | 1395 (76.4) | 29 (2.0) | 1.00 | | |
| Pre-pubertal only | 273 (14.9) | 6 (2.3) | 1.13 | 0.58, 2.22 | 0.7 |
| Pubertal onset/always | 159 (8.7) | 6 (4.0) | 1.97 | 0.87, 4.42 | 0.1 |
| Cystatin C-based eGFR (n = 2022) | | | | | |
| Never | 1544 (76.3) | 22 (1.4) | 1.00 | | |
| Pre-pubertal only | 298 (14.7) | 5 (1.7) | 1.44 | 0.67, 3.05 | 0.3 |
| Pubertal onset/always | 181 (8.9) | 8 (4.6) | 3.35 | 1.55, 7.25 | 0.002 |
| Urine albumin-creatinine ratio (n = 2141) | | | | | |
| Never | 1636 (76.4) | 47 (2.9) | 1.00 | | |
| Pre-pubertal only | 316 (14.8) | 8 (2.5) | 0.74 | 0.46, 1.19 | 0.2 |
| Pubertal onset/always | 189 (8.8) | 7 (3.6) | 1.28 | 0.63, 2.61 | 0.5 |
| Composite CKD measure (n = 1799) | | | | | |
| Never | 1374 (76.4) | 70 (5.1) | 1.00 | | |
| Pre-pubertal only | 269 (14.9) | 14 (5.0) | 0.91 | 0.60, 1.38 | 0.7 |
| Pubertal onset/always | 156 (8.7) | 15 (9.9) | 2.06 | 1.22, 3.47 | 0.007 |

Chronic kidney disease (CKD) defined as creatinine- or cystatin C-based eGFR < 60 ml/min/1.73m$^2$ or urine albumin-creatinine ratio ≥ 3.5mg/mmol.
Models adjusted for sex and age at CKD measurements.

**Table S3b. Restricted to study participants non-missing for childhood and adulthood SEP, smoking, physical activity, diabetes and hypertension.**

| Childhood overweight latent class | Model 1 | | | Model 2 | | | Model 3 | | | Model 4 | | |
|---|---|---|---|---|---|---|---|---|---|---|---|---|
| | OR | 95 % CI | *P* | OR | 95 % CI | *P* | OR | 95 % CI | *P* | OR | 95 % CI | *P* |
| Creatinine-based eGFR (n = 1304) | | | | | | | | | | | | |
| Never | 1.00 | | | 1.00 | | | 1.00 | | | 1.00 | | |
| Pre-pubertal only | 1.08 | 0.44, 2.67 | 0.9 | 1.02 | 0.42, 2.48 | 0.9 | 1.05 | 0.42, 2.60 | 0.9 | 1.09 | 0.44, 2.49 | 0.8 |
| Pubertal onset/always | 2.23 | 0.89, 5.60 | 0.09 | 2.04 | 0.83, 5.05 | 0.1 | 2.13 | 0.84, 5.42 | 0.1 | 2.31 | 0.90, 5.93 | 0.08 |
| Cystatin C-based eGFR (n = 1432) | | | | | | | | | | | | |
| Never | 1.00 | | | 1.00 | | | 1.00 | | | 1.00 | | |
| Pre-pubertal only | 1.95 | 0.72, 5.30 | 0.2 | 1.85 | 0.68, 5.01 | 0.2 | 1.93 | 0.71, 5.24 | 0.2 | 1.93 | 0.73, 5.12 | 0.2 |
| Pubertal onset/always | 5.09 | 2.05, 12.66 | <0.001 | 4.82 | 1.97, 11.80 | 0.001 | 5.00 | 1.98, 12.62 | 0.001 | 5.17 | 1.96, 13.59 | 0.001 |
| Urine albumin-creatinine ratio (n = 1453) | | | | | | | | | | | | |
| Never | 1.00 | | | 1.00 | | | 1.00 | | | 1.00 | | |
| Pre-pubertal only | 0.84 | 0.45, 1.54 | 0.6 | 0.83 | 0.45, 1.54 | 0.6 | 0.84 | 0.45, 1.54 | 0.6 | 0.86 | 0.46, 1.61 | 0.6 |
| Pubertal onset/always | 1.63 | 0.70, 3.80 | 0.3 | 1.61 | 0.69, 3.75 | 0.3 | 1.61 | 0.70, 3.73 | 0.3 | 1.67 | 0.69, 4.04 | 0.3 |
| Composite CKD measure (n = 1287) | | | | | | | | | | | | |
| Never | 1.00 | | | 1.00 | | | 1.00 | | | 1.00 | | |
| Pre-pubertal only | 0.99 | 0.57, 1.70 | 0.9 | 0.96 | 0.56, 1.64 | 0.9 | 0.97 | 0.56, 1.67 | 0.9 | 1.00 | 0.57, 1.73 | 0.9 |
| Pubertal onset/always | 2.74 | 1.53, 4.90 | 0.001 | 2.61 | 1.47, 4.65 | 0.001 | 2.69 | 1.50, 4.81 | 0.001 | 2.91 | 1.59, 5.30 | 0.001 |

Chronic kidney disease (CKD) defined as creatinine- or cystatin C-based eGFR < 60 ml/min/1.73m$^2$ or urine albumin-creatinine ratio ≥ 3.5mg/mmol.
Model 1: Adjusted for sex and age at CKD measurements.
Model 2: Adjusted for sex, age at CKD measurements and childhood and adulthood socioeconomic position.
Model 3: Adjusted for sex, age at CKD measurements and lifetime smoking trajectory.
Model 4: Adjusted for sex, age at CKD measurements and mid-adulthood physical activity trajectories.

**Table S3c. Restricted to study participants non-missing for childhood and adulthood SEP, smoking, physical activity, diabetes and hypertension.**

| Childhood overweight latent class | Model 5 | | | Model 6 | | | Model 7 | | |
|---|---|---|---|---|---|---|---|---|---|
| | OR | 95 % CI | *P* | OR | 95 % CI | *P* | OR | 95 % CI | *P* |
| Creatinine-based eGFR (n = 1304) | | | | | | | | | |
| Never | 1.00 | | | 1.00 | | | 1.00 | | |
| Pre-pubertal only | 1.03 | 0.45, 2.34 | 0.9 | 1.09 | 0.45, 2.65 | 0.8 | 0.90 | 0.41, 1.98 | 0.8 |
| Pubertal onset/always | 2.05 | 0.81, 5.22 | 0.1 | 1.96 | 0.77, 5.00 | 0.2 | 1.80 | 0.72, 4.49 | 0.2 |
| Cystatin C-based eGFR (n = 1432) | | | | | | | | | |
| Never | 1.00 | | | 1.00 | | | 1.00 | | |
| Pre-pubertal only | 1.89 | 0.68, 5.28 | 0.2 | 1.92 | 0.72, 5.14 | 0.2 | 1.68 | 0.63, 4.52 | 0.3 |
| Pubertal onset/always | 4.67 | 1.82, 12.01 | 0.001 | 4.94 | 1.99, 12.26 | 0.001 | 4.83 | 1.83, 12.73 | 0.001 |
| Urine albumin-creatinine ratio (n = 1453) | | | | | | | | | |
| Never | 1.00 | | | 1.00 | | | 1.00 | | |
| Pre-pubertal only | 0.82 | 0.45, 1.51 | 0.5 | 0.84 | 0.45, 1.56 | 0.6 | 0.87 | 0.46, 1.63 | 0.7 |
| Pubertal onset/always | 1.48 | 0.63, 3.45 | 0.4 | 1.44 | 0.62, 3.35 | 0.4 | 1.33 | 0.53, 3.33 | 0.5 |
| Composite CKD measure (n = 1287) | | | | | | | | | |
| Never | 1.00 | | | 1.00 | | | 1.00 | | |
| Pre-pubertal only | 0.95 | 0.57, 1.57 | 0.8 | 0.99 | 0.57, 1.70 | 0.9 | 0.91 | 0.54, 1.52 | 0.7 |
| Pubertal onset/always | 2.50 | 1.37, 4.56 | 0.003 | 2.50 | 1.37, 4.57 | 0.003 | 2.42 | 1.30, 4.49 | 0.005 |

Chronic kidney disease (CKD) defined as creatinine- or cystatin C-based eGFR < 60 ml/min/1.73m$^2$ or urine albumin-creatinine ratio ≥ 3.5mg/mmol.
Model 5: Adjusted for sex, age at CKD measurements and diabetes.
Model 6: Adjusted for sex, age at CKD measurements and hypertension.
Model 7: Adjusted for sex, age at CKD measurements, childhood and adulthood socioeconomic position, lifetime smoking trajectory, mid-adulthood physical activity trajectories, diabetes and hypertension.

**Table S3d. Restricted to study participants non-missing for overweight at ages 36 and 53 years.**

| Childhood overweightlatent class | Model 1 | | | Model 2 | | | Model 3 | | |
|---|---|---|---|---|---|---|---|---|---|
| | OR | 95 % CI | *P* | OR | 95 % CI | *P* | OR | 95 % CI | *P* |
| Creatinine-based eGFR (n = 1606) | | | | | | | | | |
| Never | 1.00 | | | 1.00 | | | 1.00 | | |
| Pre-pubertal only | 1.16 | 0.55, 2.42 | 0.7 | 1.00 | 0.49, 2.04 | 0.9 | 1.10 | 0.53, 2.28 | 0.8 |
| Pubertal onset/always | 1.90 | 0.79, 4.56 | 0.2 | 1.17 | 0.50, 2.75 | 0.7 | 1.63 | 0.68, 3.89 | 0.3 |
| Cystatin C-based eGFR (n = 1771) | | | | | | | | | |
| Never | 1.00 | | | 1.00 | | | 1.00 | | |
| Pre-pubertal only | 1.75 | 0.77, 3.99 | 0.2 | 1.52 | 0.66, 3.53 | 0.3 | 1.60 | 0.69, 3.70 | 0.3 |
| Pubertal onset/always | 3.80 | 1.63, 8.88 | 0.002 | 2.42 | 0.98, 5.95 | 0.05 | 2.97 | 1.28, 6.94 | 0.01 |
| Urine albumin-creatinine ratio (n = 1867) | | | | | | | | | |
| Never | 1.00 | | | 1.00 | | | 1.00 | | |
| Pre-pubertal only | 0.78 | 0.48, 1.27 | 0.3 | 0.72 | 0.44, 1.19 | 0.2 | 0.76 | 0.46, 1.24 | 0.3 |
| Pubertal onset/always | 1.35 | 0.65, 2.80 | 0.4 | 1.07 | 0.50, 2.28 | 0.9 | 1.26 | 0.60, 2.65 | 0.5 |
| Composite CKD measure (n = 1584) | | | | | | | | | |
| Never | 1.00 | | | 1.00 | | | 1.00 | | |
| Pre-pubertal only | 0.97 | 0.62, 1.50 | 0.9 | 0.85 | 0.55, 1.32 | 0.5 | 0.93 | 0.60, 1.45 | 0.8 |
| Pubertal onset/always | 2.10 | 1.21, 3.63 | 0.008 | 1.42 | 0.81, 2.49 | 0.2 | 1.90 | 1.09, 3.30 | 0.02 |

Chronic kidney disease (CKD) defined as creatinine- or cystatin C-based eGFR < 60 ml/min/1.73m$^2$ or urine albumin-creatinine ratio ≥ 3.5mg/mmol.
Model 1: Adjusted for sex and age at CKD measurements.
Model 2: Adjusted for sex, age at CKD measurements and overweight at age 36 years.
Model 3: Adjusted for sex, age at CKD measurements and overweight at age 53 years.
