# Supplementary material for: Early-Life Overweight Trajectory and CKD in the 1946 British Birth Cohort Study
Source: Am J Kidney Dis. 2013 Aug;62(2):276–84. doi: 10.1053/j.ajkd.2013.03.032 (PMC3719096; doi:10.1053/j.ajkd.2013.03.032)
Supplement: Supplementary Table S4 (PDF) — Linear regression coefficients for cystatin C level at age 60-64 by early-life overweight latent class, by complete case analyses. [file mmc4.pdf]

Table S4. Linear regression coefficients (coeff) for cystatin C at age 60-64 years by early-life overweight latent class.

Table S4a.

Childhood overweight latent class	n (%) in this latent class	Mean (SD) cystatin C (mg/l)	Coeff	95 % CI	*P*
Cystatin C (mg/l) (n = 2022)					
Never	1544 (76.3)	0.819 (0.142)	(ref)		
Pre-pubertal only	298 (14.7)	0.823 (0.146)	0.001	-0.017, 0.019	0.9
Pubertal onset/always	181 (8.9)	0.864 (0.191)	0.045	0.022, 0.067	<0.001

Models adjusted for sex and age at cystatin C measurement.

Table S4b. Restricted to study participants non-missing for childhood and adulthood SEP, smoking, physical activity, diabetes and hypertension.

Childhood overweight latent class	Model 1			Model 2			Model 3			Model 4		
	Coeff	95 % CI	*P*	Coeff	95 % CI	*P*	Coeff	95 % CI	*P*	Coeff	95 % CI	*P*
Cystatin C (mg/l) (n = 1432)												
Never	(ref)			(ref)			(ref)			(ref)		
Pre-pubertal only	0.001	-0.021, 0.023	0.9	-0.001	-0.023, 0.022	0.9	0.001	-0.021, 0.023	0.9	0.002	-0.020, 0.024	0.9
Pubertal onset/always	0.051	0.024, 0.078	<0.001	0.048	0.022, 0.075	<0.001	0.050	0.024, 0.077	<0.001	0.052	0.026, 0.079	<0.001

Model 1: Adjusted for sex and age at CKD measurements.
Model 2: Adjusted for sex, age at CKD measurements and childhood and adulthood socioeconomic position.
Model 3: Adjusted for sex, age at CKD measurements and lifetime smoking trajectory.
Model 4: Adjusted for sex, age at CKD measurements and mid-adulthood physical activity trajectories.

Table S4c. Restricted to study participants non-missing for childhood and adulthood SEP, smoking, physical activity, diabetes and hypertension.

Childhood overweight latent class	Model 5			Model 6			Model 7		
	Coeff	95 % CI	*P*	Coeff	95 % CI	*P*	Coeff	95 % CI	*P*
Cystatin C (mg/l) (n = 1432)									
Never	(ref)			(ref)			(ref)		
Pre-pubertal only	0.001	-0.021, 0.023	0.9	0.001	-0.021, 0.023	0.9	0.000	-0.022, 0.022	0.9
Pubertal onset/always	0.048	0.021, 0.075	<0.001	0.050	0.023, 0.076	<0.001	0.048	0.022, 0.075	<0.001

Model 5: Adjusted for sex, age at CKD measurements and diabetes.
Model 6: Adjusted for sex, age at CKD measurements and hypertension.
Model 7: Adjusted for sex, age at CKD measurements, childhood and adulthood socioeconomic position, lifetime smoking trajectory, mid-adulthood physical activity trajectories, diabetes and hypertension.

Table S4d. Restricted to study participants non-missing for overweight at ages 36 and 53 years.

Childhood overweight latent class	Model 1			Model 2			Model 3		
	Coeff	95 % CI	*P*	Coeff	95 % CI	*P*	Coeff	95 % CI	*P*
Cystatin C (mg/l) (n = 1771)									
Never	(ref)			(ref)			(ref)		
Pre-pubertal only	0.001	-0.019, 0.021	0.9	-0.003	-0.023, 0.017	0.8	-0.001	-0.021, 0.018	0.9
Pubertal onset/always	0.042	0.019, 0.066	0.001	0.028	0.003, 0.053	0.02	0.036	0.011, 0.060	0.004

Model 1: Adjusted for sex and age at CKD measurements.
Model 2: Adjusted for sex, age at CKD measurements and overweight at age 36 years.
Model 3: Adjusted for sex, age at CKD measurements and overweight at age 53 years.
