# Supplementary material for: Early-Life Overweight Trajectory and CKD in the 1946 British Birth Cohort Study
Source: Am J Kidney Dis. 2013 Aug;62(2):276–84. doi: 10.1053/j.ajkd.2013.03.032 (PMC3719096; doi:10.1053/j.ajkd.2013.03.032)
Supplement: Supplementary Figure S1 (PDF) — Flow diagram showing inclusion in early-life overweight latent class analysis and multiple imputation analysis. [file mmc5.pdf]

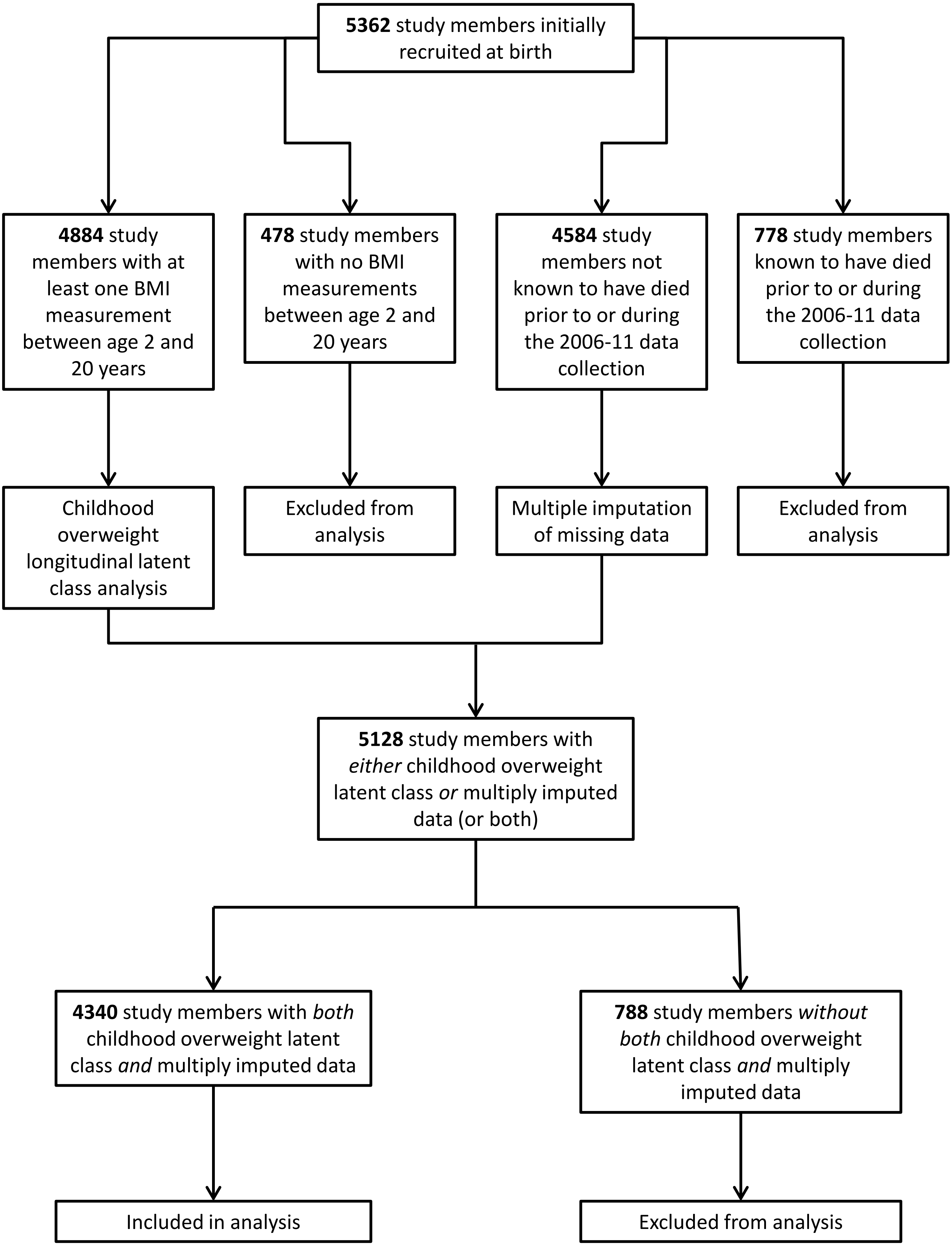

Figure S1: Flow diagram showing inclusion in early-life overweight latent class analysis and multiple imputation analysis.
